# Exosomes in craniofacial tissue reconstruction

**DOI:** 10.1186/s40902-022-00357-3

**Published:** 2022-08-24

**Authors:** Muhja Salah, Farhad B. Naini

**Affiliations:** 1 Centre for Additive Manufacturing, University of Nottingham, Nottingham, UK; 2 Kingston and St George’s University Hospitals, London, UK

**Keywords:** Bone tissue engineering, Mesenchymal stem cells, Exosomes, Biogenesis and tissue bone scaffolds

## Abstract

**Background:**

Mesenchymal stem cell (MSC) therapy gained interest among scientists following the discovery of its therapeutic potential. However, their clinical use has been hindered due to their immunogenicity and tumorigenicity. Relatively recently, it has been unveiled that the mechanism by which MSC promote healing is by secreting exosomes. This raised the interest in developing cell-free therapy, avoiding the obstacles that deterred the translation of MSC therapy into clinical practice.

**Review:**

This comprehensive narrative review summarises the current understanding of exosome biogenesis and content. Moreover, the existing research on exosome use in bone tissue engineering is discussed.

**Conclusions:**

Exosome-based therapy may provide excellent potential in the field of bone tissue engineering and craniofacial reconstructive surgery. Further investigation is required before the technology can be translated into clinical practice.

## Background

Bone grafts used in craniofacial surgery provide the osseoconduction, which is the frame over which bone-generating cells will adhere to promote bone formation and healing and stimulate the cells to differentiate into osteoblasts and form bones, which is referred to as osseoinduction. Grafts can be allogeneic, meaning that they can be collected from other patients, but this can elicit an immune reaction or cause transmission of disease [1]. To eliminate the immunogenicity of an allograft, they are frozen, freeze-dried or irradiated to promote their sterility, which can also decrease their osseoconductivity and osseoinductivity [2]. Alternatively, a graft can be autologous, collected from the same patient from a donor site, such as the patient’s rib, femur or iliac crest. This eliminates the complication associated with allogeneic graft use. However, it has the disadvantage of adding a second site of surgery, which increases patient morbidity and the possibility of infection, haemorrhage or nerve damage. It also has the disadvantage of harvesting only a limited amount of the bone that might not be suitable when a more significant amount of the bone is needed. Thus, finding alternatives to bone grafting has gained research interest in the last three decades, which has led to the development of bone tissue engineering research focused on finding alternatives to bone grafting and enhances bone regeneration via a combination of biomaterials, cells and growth factors [3].

Since the development of stem cell biology and the discovery that they are unspecialised cells with the multipotential to differentiate into many different cell types, it became evident that they are the best choice of cells to enhance tissue regeneration, including the bone [4]. Mesenchymal stem cells (MSC) are easier to harvest as they are found in many sites in the body. Their use also negates the ethical hurdle when using embryonic stem cells, which necessitate killing an embryo in order to obtain the cells [5]. They proved efficacy in treating a non-union fracture. In 2017, Emadedin et al. reported the safety and efficacy of injecting one-time implantation of 20–50 × 106 MSCs into non-union sites and followed patients up for a year with radiographs [6]. Similarly, Castillo-Cardiel et al. reported that treating mandibular fracture using adipose-derived MSC resulted in a higher ossification rate than the control group who received only reduction and fixation [7]. Moreover, stem cells combined with bone graft proved efficient in treating and repairing bone gaps caused by defects. Khojasteh et al. reported that using buccal fat pad-derived MSC and lateral ramus cortical plate to repair alveolar clefts exhibited higher bone formation after 6 months from the operation [8].

Despite the reported success associated with cell-based therapy in repairing bone, it has still faced several limitations from immunogenicity, tumorigenicity and low survival rate that have hindered its progress [9]. However, recent studies have suggested that MSC do not regenerate by engraftment into tissue. Instead, they secrete various cytokines, growth and angiogenic factors that cause tissue regeneration, angiogenesis and immune modulation [10]. These secreted factors are contained within an extracellular vesicle (EV) or exosomes. This has provided the impetus for research in their use as a cell-free alternative to the MSC regenerative treatment approach.

An early investigation by Gnecchi et al. in 2005 found that injecting a myocardial infarction site with MSC enhanced cardiac repair and function, which occurred in less than 72 hours [11]. They postulated that this early recovery effect does not occur due to MSC differentiation into cardiomyocytes but due to the early release of paracrine factors from the MSC. They proved their hypothesis by preparing conditioned media from the MSC (which contained the exosomes) and subjected adult rat ventricular cardiomyocytes (ARVC) into hypoxic conditions to resemble infarction, and when cultured those cells in the conditioned media, they found an increase in the number of ARVC compared to the control group. Further studies showed that exosomes function similar to the proposed MSC function by increasing proliferation of the target tissue, preventing apoptosis and aiding regeneration by allowing immunomodulation and increasing vascularisation [12]. From that study, the cell-free approach gained more interest as it presents a similar advantage for tissue regeneration. At the same time, it guarantees low immunogenicity since exosomes lack biological markers responsible for eliciting an immune response, along with the shielding effect provided by the membrane structure surrounding the exosome that prevents it from degradation [13].

### Exosome biogenesis

**Fig. 1:**
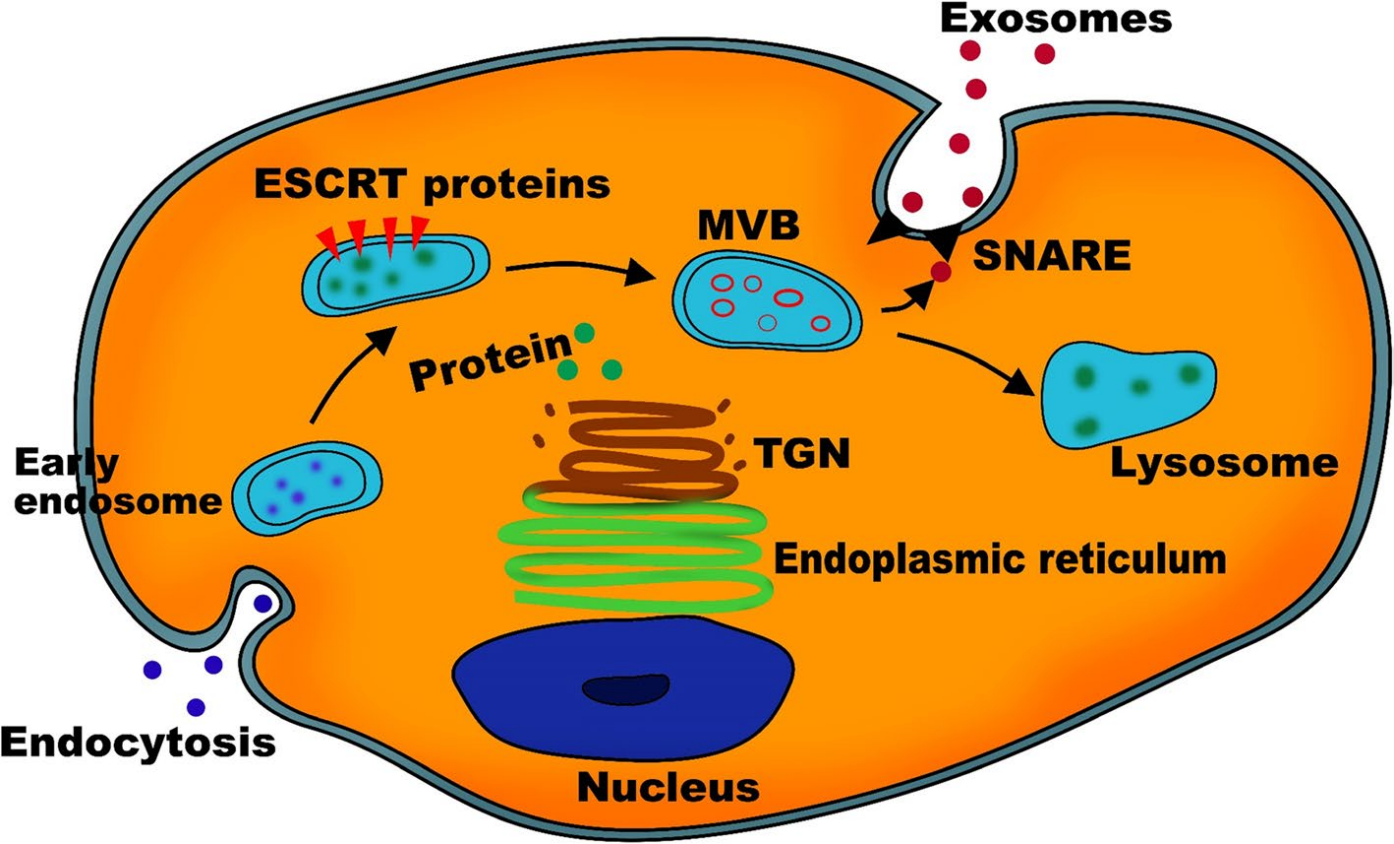
Exosome biogenesis starts with the endocytosis and early endosome (EE) formation. In addition to extracellular cargo that gets to the EE, it receives cargo and protein from the trans-face of the Golgi network (TGN). The cargo undergoes meticulous surveillance and multivesicular body (MVB) containing the intraluminal vesicles (ILV). The cargo that should be degraded is trafficked toward the lysosome while the exosomes are carried on SNARE proteins to help it dock on the cytoplasmic membrane to be excreted as exosomes

Exosomes are membrane-bound intraluminal cell vesicles of 40–100 nm width that bound the plasma membrane to be excreted with its cargo content into the extracellular space by all cell types [14, 15]. The importance of these exosomes stems from the fact that it is a way of communication and crosslinking between cells or even distant tissues [16]. Moreover, as they contribute to cells and tissues in healthy conditions, they are also attributed to the development and progression of diseases [17]. Their effect is mediated by their cargo content, which is heterogeneous, loaded with active molecules including lipids, nucleic acid or proteins. This cargo is carried inside the exosome until reaching the recipient cell to induce a signalling molecule that changes its physiological process [18]. As they pass outside the cells, exosomes escape phagocytosis by phagocytic cells. Moreover, when internalised into the target cell, they circumvent lysosomes and their degradation effect [19]. Exosome biogenesis starts by internalising some extracellular fluid and macromolecules by endocytosis, which entails inward budding of the plasma membrane [20]. The process enables the cells to identify and adapt to changes in their environment. The endocytosis process produces the early endosome (EE). EE also receives cargo from the Trans-face of the Golgi Network (TGN), a vital cell organelle involved in packaging proteins after their production in the exosomes. EE undergoes a maturation process in which it recycles its content by activation, silencing and degrading and eventually sorting the content or cargo to turn into a late endosome (LE) [21]. EE tagged with ubiquitylated proteins in their cytosol attract the Endosomal Sorting Complex Required for Transport (ESCRT), sorting machinery needed to mature the EE into LE. ESCRT complexes are a group of four proteins (ESCRT-0 to ESCRT-3) found in the cell cytosol, which gets attracted to the ubiquitin tag on the intracellular part of the membrane proteins. They work in sequential order as activation of ESCRT-0 leads to activation of ESCRT-1 and so forth. ESCRT-0 and ESCRT-1 are involved in identifying the ubiquitin tag on the cargo. In contrast, ESCRT-2 and 3 are involved in sorting the cargo and producing invaginations to build walls between the sorted cargo and producing intraluminal vesicles (ILV) within a multivesicular body (MVB) (Fig. 1) [22].

MVB formation is a crucial step in exosome biogenesis, which is considered a large vesicle containing multiple smaller vesicles within it. Following MVB formation, they fuse with lysosomes when their cargo is destined to be degraded. Otherwise, they connect to the plasma membrane, where ILV is released into the extracellular fluid as exosomes [23]. MVB transportation into their final destination (lysosome or plasma membrane) depends on the contraction of the molecular motor proteins (myosin and actin) responsible for cell motility, organelle movement and cell mitosis. Following their docking on the plasma membrane from the cytosol side, another protein called SNARE (soluble NSF-attachment protein surface) is responsible for fusing the plasma membrane to the MVB membrane and causing their secretion into the extracellular environment [24].

### Exosome content

Exosome contents differ according to the cells secreting them. They share specific proteins such as those vital for MVB biogenesis and release (e.g. Alix, TSG101, Clathrin). They also share the proteins responsible for transport and fusion with the cell membrane (e.g. annexins and Rab), and cytoskeleton proteins needed for movement (e.g. actin and tubulin), heat shock proteins, which are involved in stress response (HSP70 and HSP90), and tetraspanin proteins (CD9, CD81 and CD82), which affect the exosome membrane and help in budding and cell penetration [25–27]. Shared proteins are commonly used as markers to identify and characterise exosomes as ALIX, TSG101 and different tetraspanins [28]. Other proteins in exosomes are related to the cells from which they are excreted.

Alongside proteins, exosomes are rich with different types of ribonucleic acids (RNA), including microRNA (miRs), which is the most abundant type alongside a few percentages of other types of RNAs, such as the ribosomal RNA (rRNA), messenger RNA (mRNA), long non-coding RNA (lncRNA) and other non-coding RNAs [29, 30]. MiRs are made of 22 nucleotides on average, which are considered non-coding, meaning that they are not translated into proteins. Alternatively, they have a function regulating the gene expression or silencing [31]. MiR-214, miR-29a, miR-1, miR-126 and miR-320 are among the discovered miRs that fill exosomes and function in health and disease regulating angiogenesis, oncogenesis and haematopoiesis. Wang et al. in a comprehensive animal model study found that increasing the expression of miR-29 decreases fibrosis in kidney proximal tubular epithelial cells by regulating collagen expression [32]. In contrast, Kogure et al. found that lncRNA TUC399 transferred between cells within exosomes was implicated in the growth and spread of hepatocellular cancer [33]. Thus, they considered that lncRNA-loaded exosomes are a signalling mechanism involved in tumour growth by influencing genetic expression on cells within the microenvironment. Similarly, in 2015, Conigliaro et al. reported that exosomes excreted from CD90 + cells are rich with lncRNA H19 that is found to influence angiogenesis and release of vascular endothelial growth factor (VEGF), thus promoting tumour development [34]. Nowadays, miRNA is considered a target for cancer therapy with many drugs, whether they are miRNA inhibitors or miRNA mimics is currently under investigation [35].

Besides RNAs, exosomes contain deoxyribonucleic acid (DNA), either derived from the genome or the mitochondria. DNA gets secreted passively after cell death or actively by live cells in health and disease by breaking in the nucleus during cell division or caused by a damage mechanism [36]. The resultant is a polynucleosome (a variant number of nucleosome) which is either in the form of a double-strand (ds), single-strand (ss), a mitochondrial fragment if originated from mitochondria (mt) or a circular DNA fragment secreted from the cell as a cell-free DNA (cfDNA). Fernando et al. analysed the plasma cfDNA concentration and showed that around 93% of it is located in exosomes which render it stable protected from degradation or attack from the immune system [37, 38]. Exosomal DNA has many biological functions to the recipient cells, modulating them and regulating the immune system [36].

Once the exosome is shed to the extracellular environment, it gets transported into its target cell. Different labelling techniques have been used to examine the biodistribution of exosomes, including bioluminescence imaging, magnetic resonance imaging and others [39]. It was found that the exosome journey from the mother cell to its target cell depends on their cell source, their size as well as the surface proteins present, but, with a large number of exosomes being trapped in the liver, spleen, lungs, bone and lymph nodes [40]. Understanding the factors affecting exosome trafficking allows exploiting their therapeutic potential and increases safety. Adding moieties, protein surfaces or lipids are used either to enhance the natural targeting mechanism of exosome biodistribution or as bioengineered targets that will manipulate and help exosomes identify specific target cells [40]. Upon reaching its target cell, exosomes fuse to the plasma membranes of the target cell and get internalised via endocytosis to release their content into the cytosol [13]. Alternatively, they dock on a transmembrane ligand to induce downstream signal transduction that activates the cell [41].

### Osteoblast exosomes

Several studies were conducted to analyse the exosome content secreted directly by osteoblasts. In 2007, Xiao et al. published their proteomic analysis report. They identified many proteins within osteoblastic exosomes such as bone morphogenetic protein (BMP), alkaline phosphatase (ALP), eukaryotic initiation factor 2 (eIF2), osteopontin (OPN), osteocalcin (OCN) and osteonectin (ON) secreted by osteoblasts to enhance mineralisation [42]. Huynh et al. characterised receptor activator of nuclear factor kappa-B (RANKL) as a component of osteoclast exosome content essential for osteoclastogenesis [43]. Liu et al. undertook an extensive review of the role of exosomes in bone remodelling, finding that exosomes deliver different miRNAs, such as miR-214-3p, miR-183-5p and miR-196a, as well as other growth factors (e.g. BMP and TGF_β1_), that regulate bone formation [44].

### Mesenchymal stem cell exosomes (MSC-Exos)

In 2010, Lai et al. were the first research group to isolate exosomes from the bone marrow-derived MSC and showed their ability to reduce myocardial infarction in mouse models [45]. Their work supported the hypothesis that the MSC produces their regenerative effect by a paracrine effect through release of cytokines and growth factors to promote regeneration instead of only differentiating into the cell lineage they are repairing. Following their success, other studies demonstrated MSC-exos ability in promoting tissue regeneration. Zhang et al. facilitated cutaneous wound healing [46], Bruno et al. improved the recovery from acute kidney injury [47], Tan et al. elicited a hepatoprotective effect against induced liver injury models [48], Zhang et al. promoted axonal regeneration [49], Zhang et al. alleviated TMJ osteoarthritis [50] and Zhang et al. promoted bone regeneration via combining exosomes into a tricalcium phosphate scaffold [51].

MSC therapy had proved to be efficient in many clinical trials. However, many risks existed with MSC therapy, such as eliciting an immune response, potential tumorigenicity and genetic instability [52]. Using the cell-free approach with exosomes derived from MSC is considered safer, avoiding the risks and allowing for the repeat administration of the therapy without the fear of MSC impaction in non-targeted tissues, especially the lungs [53]. Its production is also considered easier and more cost-effective than stem cell therapy, as with ultracentrifugation, a large scale of exosomes can be produced from a specific cell line [54]. Besides all these benefits, exosomes can be engineered to become carriers for drug delivery or small molecules, which has the advantage of decreasing the drug dosage and minimising the anticipated side effects [55]. To date, a number of studies have assessed the effect of MSC-derived exosomes on bone modelling and regeneration. Lu et al. found the exosomes derived from adipose tissue-MSC preconditioned with TNF-α promoted osteodifferentiation by inhibiting Wnt signalling [56]. Zhao et al. also showed that coculturing MSC-exos with an osteoblast cell line increased the proliferation of the osteoblasts cells through mitogen-activated protein kinase (MAPK) signalling, which is essential in the cell cycle and growth [57]. Another pathway for suppressing bone healing was found by Xu et al. which is the micro-RNA (miR-128-3P) specifically carried by aged MSC-exos. The group hypothesised that using an anti-(miR-128-3P) could be a target for bone healing, especially in the elderly [58]. These studies and others, in aggregate, show that MSC-exos could be a potential therapeutic tool in treating bone, fractures and bone tissue engineering.

### Bone tissue engineering (BTE) and exosomes

Bone tissue engineering is a growing field aiming to produce scaffolds from biocompatible, bioactive material that is seeded with MSC for their ability to differentiate down the osteogenic pathway and immunoregulation to promote healing and bone repair. However, with the rising trend toward non-cell therapy, exosomes can represent an alternative to MSC for bone regeneration. Li et al. fabricated scaffolds with polylactic co glycolic acid (PLGA) and combined them with exosomes derived from adipose tissue-derived MSC [59]. They showed that the exosomes’ presence in the scaffold greatly enhanced bone repair in animal models compared to the PLGA scaffold alone. In the same direction, Zhang et al. found that seeding a scaffold made from tricalcium phosphate (TCP) with exosomes derived from human-induced pluripotent stem cell-derived mesenchymal stem cell (hiPS-MSC-exos) are able to repair critical-size calvarial defects in rat models through the activation of the PI3K/Akt signalling pathway which is essential for cell proliferation, metabolism, survival, growth and angiogenesis [51]. Xie et al. used decalcified bone extracellular matrix (dECM) for scaffold fabrication and compared the osteogenic potential of these bare scaffolds with scaffold coated the MSC-exos using micro-computed tomography analysis and histological analysis [60]. They found that using exosomes greatly enhanced vascularisation of the scaffolds, thus promoting osteogenesis. In a unique recent study, Wu et al. added neuromodulation cues to angiogenesis in its importance for bone regeneration. In their study, they used Schwann cell (SC) exosomes and cultured them with bone marrow stromal cells (BMSC) in vitro and observed the enhancement of osteogenic differentiation of the BMSC [61]. They again used the combination to coat porous titanium (Ti) scaffolds and showed that SC-derived exosomes greatly enhanced the biological activity of the Ti scaffold. Diomede et al. used a 3D printing technology to fabricate scaffolds made from polylactic acid (PLA) polymer, seeded the gingival-derived MSC-derived exosomes and implanted them in animal model calvarial defects [62]. The group further compared using the 3D-printed PLA scaffold seeded with regular exosomes with engineered exosomes using polyethyleneimine (PEI) and found that the PEI-exos showed greater osteogenic inductivity and better healing in the animal models. Gandolfi et al. took Diomede’s experiment further. They produced PLA scaffolds and doped the surface with calcium silicate (CaSi) and dicalcium phosphate dihydrate (DCPD) and measured its effect on adipose-derived MSC [63]. They found that the dope layer enhanced bone repair as the calcium and silicon ions stimulated mineralisation.

## Conclusions

There is a definitive need for investigating alternatives to the bone graft. Morbidity of autogenous bone grafts, the limited amount of the bone to be harvested and the need to have grafts that can enhance the aesthetic outcome of the surgery have increased the research in bone tissue engineering. Since exosomes are extracted from MSC and carry the same therapeutic potential, the cell-free approach has gained much interest. It provides intracellular communication needed for tissue regeneration through the regulation of cytokines, facilitating angiogenesis and osteoblast differentiation and mineralisation. Moreover, all its bioactive contents from proteins, microRNAs and DNAs are enveloped within the lipid bilayer, are stable and are protected from degradation. They could be seeded in scaffolds for tissue regeneration instead of seeding scaffolds with cells. Although exosomes are considered a powerful treatment tool, the technology is still new. There should be a consensus on the method of purification and isolation as well as the effective concentration. Furthermore, and with the advances in biomaterials used for scaffold design, further investigations need to be carried out to identify the best combination without affecting the exosome potential. In summary, exosome-based therapy may provide excellent potential in the field of bone tissue engineering and for craniofacial reconstructive surgery. However, further investigation is required before the technology can be translated into clinical practice.

## Data Availability

Not applicable.

## Abbreviations

ALP: Alkaline phosphatase
ARVC: Adult rat ventricular cardiomyocytes
BMP: Bone morphogenic protein
BMSC: Bone marrow stem cell
CaSi: Calcium silicate
cfDNA: Cell-free DNA
DCPD: Dicalcium phosphate dihydrate
dECM: Decellularized extracellular matrix
DNA: Deoxyribonucleic acid
EE: Early endosome
eIF2: Eukaryotic initial factor 2
ESCRT: Endosomal sorting complex requirement for transport
Exos: Exosome
ILV: Intraluminal vesicle
LE: Late endosome
lnc-DNA: Long non-coding DNA
MAPK: Mitogen-activated protein kinase
miR: Micro-RNA
mRNA: Messenger RNA
MSC: Mesenchymal stem cell
mt: Mitochondria
MVB: Multivesicular body
OCN: Osteocalcin
ON: Osteonectin
OPN: Osteopontin
PEI: Polyethyleneimine
PLGA: Polylactic co glycolic acid
RANKL: Receptor activator of nuclear factor kappa-B
RNA: Ribonucleic acid
rRNA: Ribosomal RNA
SC: Schwann cell
SNARE: Soluble NSF-attachment protein surface
TCP: Tricalcium phosphate
VEGF: Vascular endothelial growth factor
